# Microfluidic model of the platelet-generating organ: beyond bone marrow biomimetics

**DOI:** 10.1038/srep21700

**Published:** 2016-02-22

**Authors:** Antoine Blin, Anne Le Goff, Aurélie Magniez, Sonia Poirault-Chassac, Bruno Teste, Géraldine Sicot, Kim Anh Nguyen, Feriel S. Hamdi, Mathilde Reyssat, Dominique Baruch

**Affiliations:** 1 PlatOD, Paris, France; 2 MMN, UMR CNRS 7083 Gulliver, ESPCI Paris, PSL Research University, 10 rue Vauquelin, 75005 Paris, France; 3 Sorbonne Universités, Université de Technologie de Compiègne, UMR CNRS 7338, BMBI, Compiègne France; 4 Inserm, UMR_S1140, Paris, France; 5 Inserm, UMR_S1148, Paris, France

## Abstract

We present a new, rapid method for producing blood platelets *in vitro* from cultured megakaryocytes based on a microfluidic device. This device consists in a wide array of VWF-coated micropillars. Such pillars act as anchors on megakaryocytes, allowing them to remain trapped in the device and subjected to hydrodynamic shear. The combined effect of anchoring and shear induces the elongation of megakaryocytes and finally their rupture into platelets and proplatelets. This process was observed with megakaryocytes from different origins and found to be robust. This original bioreactor design allows to process megakaryocytes at high throughput (millions per hour). Since platelets are produced in such a large amount, their extensive biological characterisation is possible and shows that platelets produced in this bioreactor are functional.

Platelets are small anucleate cells that circulate in blood and are responsible for the arrest of bleeding. Platelets are formed by fragmentation of larger cells called megakaryocytes (MKs). Thrombocytopenia (insufficient platelet count) is a major condition, often requiring platelet transfusions. High collection costs, donor shortage, immunogenicity, risk of contamination and storage issues represent the main limits of this therapeutic approach. People have tried to make artificial platelets but these objects seem promising for drug targeting rather than for therapy^1^. Recently, several groups have managed to produce platelet-generating MKs from stem cells, either embryonic^2^ or induced pluripotent stem cells^3^. Cell culture techniques have improved in order to reduce product immunogenicity^4^. This constitutes a very promising perspective as an alternative to platelet donation and transfusion. However, one of the main challenges for *in vitro* production remains the production yield, as each transfusion requires 1 to 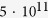 platelets^5^.

Compared with static culture, platelet production can be accelerated by exposing MKs to shear and von Willebrand factor (VWF)^6^. Intravital microscopy in mice skull revealed that bone marrow MKs produce extensions into microvessels whose flow-induced ruptures yield platelets^7^. Several attempts have been made to mimic the osteoblastic niche with a microfluidic environment^3^,^8^,^9^,^10^. These systems rely on porous membranes separating a MK reservoir from a channel where elongations are subjected to shear. Although these systems lead to MK fragmentation, crawling through a narrow pore is a slow process^10^. Producing billions of platelets with this technology would require a high degree of parallelisation that has not been achieved yet^8^. To overcome these limitations, we build a microfluidic system where MKs are directly injected into microchannels comprising arrays of VWF-coated pillars acting as anchors. In this paper we describe the capture, elongation and fragmentation of MKs on hundreds of thousands pillars. Our setup allows to visualise the entire platelet formation process, from the initial capture of a flowing MK upon its encounter with a pillar to the formation of elongations and finally to the release of (pro)platelets in the flowing media. Platelets are collected, counted and characterised. We demonstrate that microchannels textured with organised micropillar arrays are able to convert MKs into platelets with high efficiency, and to process large amounts of cells, contrary to biomimetic membrane-based bioreactors.

## Results

### Perfusion of MKs through a 3D micropillar forest

**Figure 1:**
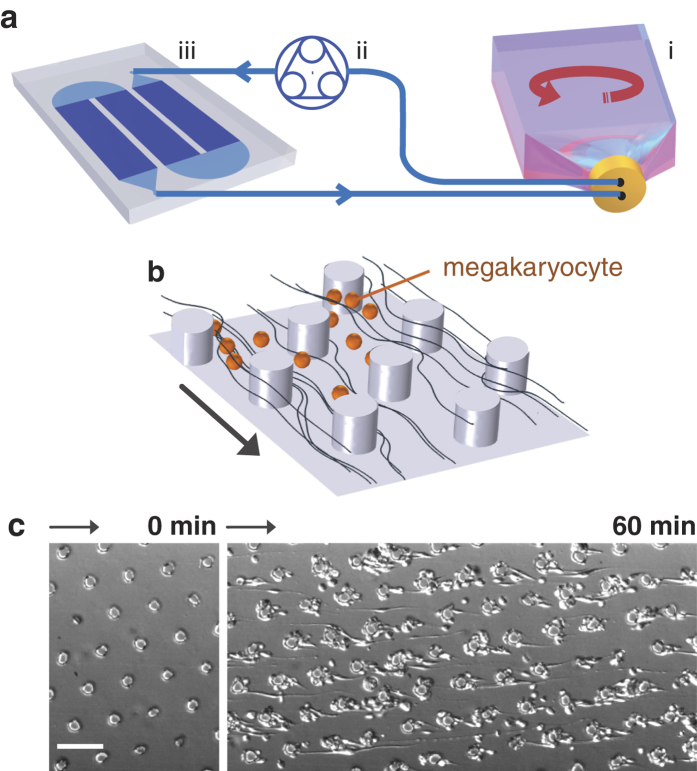
Design and principle of the experiment. (**a**) Sketch of the experimental setup, including a container for cell suspension placed on an orbital mixer (i) to keep the cell concentration uniform, a peristaltic pump (ii), the microfluidic production device (iii), and tubing. The microfuidic device consists in a large entrance compartment, distributing the suspension among sixteen straight channels, whose serpentine shape allows the design to fit in a single glass slide. Each of these channels is 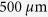 wide, 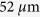 deep and 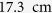 long. The dark blue region corresponds to the pillar forests that cover the straight part of the channels, while the U-turns are devoid of obstacles. (**b**) Pillars are arranged on a tilted hexagonal lattice. The angle between the main axis of the channel and that of the lattice is fixed to 10° in order to ensure that each cell encounters at least one pillar. The streamlines are illustrated with black lines and MKs with orange spheres. (**c**) Top view of the micro pillar array before cell perfusion and after 60 minutes perfusion. The capture process of cells at intermediate times will be described in Fig. 2. Scale bar, 100 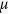
m.

When perfused in a VWF-coated smooth channel under high shear rates (>1800 s^−1^ at the walls), MKs adhere to the walls where they roll, elongate and break into platelets^6^. In the present work, we amplify this capture effect by increasing the surface/volume ratio of the channel. We design a flow chamber composed of parallel channels in which pillars are arranged on an array (Fig. 1). The VWF-coated pillar array is tilted from the mean flow direction to promote interaction between flowing MKs and pillars (Fig. 1c). Simulations show that the wall shear rate ranges from 0 to 5000 s^−1^ at the lateral surface of pillars (supplementary Fig. S1).

In these obstacle filled channels, the number of cells arrested at the solid surface exceeds by far the numbers attained in a smooth channel, and increases with pillar density (supplementary Fig. S2a). When the distance between two pillars or between the top and bottom surfaces of the channel decreases and becomes comparable to the size of cells, MK density gradually increases on the pillars and channel solid surface, until forming clogs (supplementary Fig. S2b et S2c).

MK density can thus be tuned using the micropillar forest geometry. Unless otherwise stated, experiments presented in the next section are performed with a cell concentration of 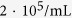 and a centre-to-centre distance 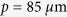 between pillars. In these conditions, we observe enhanced capture compared with a smooth channel, but cell density remains low enough so that the observation of individual cells remains possible.

### Cell capture and elongation

**Figure 2:**
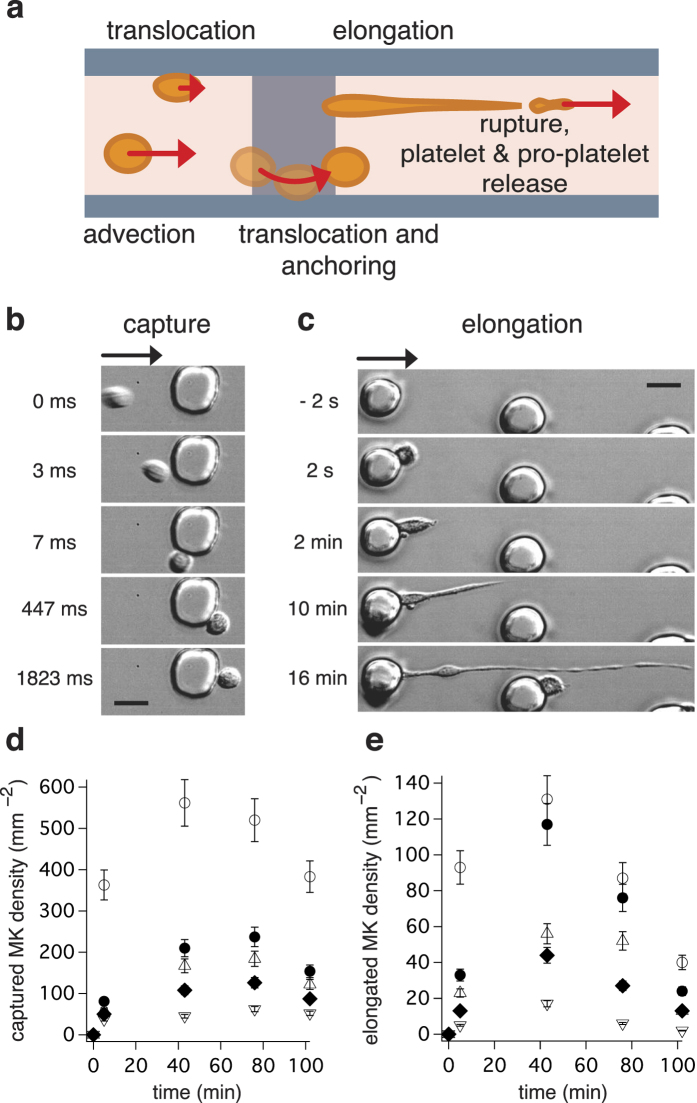
Capture and elongation of MKs on pillars. (**a**) Side view representing the possible behaviours of cells in the chip: advection, translocation along the smooth surface of the channel, translocation along the sides of pillars, and capture followed by elongation. (**b**) Chronophotography of a MK capture: The advected MK is captured at 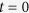 then translocates around a pillar. In advection it takes 3 ms for the MK to move 3 times its diameter, while 1.8 s are necessary to roll over the same distance by translocation. Scale bar, 20 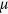
m. (**c**) Top view of a MK elongation. The montage starts at −2 s, before the MK is captured by the left pillar. Scale bar, 20 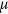
m. (**d**) Surface density of captured MKs as a function of time during a perfusion of 6,7 ml at 200 000 MK/mL suspension through 1 chip, measured at different positions: 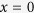 mm (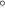), 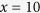 mm 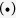, 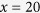 mm 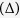, 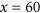 mm 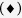, 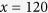 mm (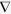). Counts were performed by two experimentalists and represented as (mean ± SD). (**e**) Surface density of elongated MKs as a function of time for the same conditions.

Perfused MKs have a diversity of behaviours depicted in Fig. 2a. They may remain in advection, be captured at the surface of a pillar or at the wall and translocate, which may be followed by elongation and rupture into proplatelets and platelets. Among these behaviours, some have already been observed, such as translocation along selectin-coated pillars^11^ and permanent capture by ligand-coated surfaces. Figure 2b illustrates the dramatic deceleration of a MK encountering a pillar. In a few milliseconds it gets captured at the VWF surface and then translocates along the side surface. In a few seconds it reaches the downstream side of the pillar, where it can stay attached for a much longer time (over 15 minutes), as shown in Fig. 2c and Supplementary Video 1. Once captured, MKs change their shape, as will be discussed later in the manuscript. Figure 2d shows a typical curve representing the density 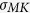 of MKs captured at the surface of walls and pillars. The capture process is not linear: the number of trapped MKs increases very fast during the first 30 minutes of perfusion, then seems to saturate and even decrease. In experiments where perfusions lasted more than 2 hours, we even observed a decrease of 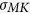. In Supplementary Fig. S6 we also show that capture is enhanced by the presence of VWF. In a BSA-coated bioreactor, although some cells encounter pillars and might get stuck at their surface, few of them elongate. Instead of promoting lateral translocation, hydrodynamic forces here either squeeze the cell against the pillar or detach it from a pillar.

After one hour of perfusion, each pillar may have captured one or several cells (Fig. 1c). A few minutes after anchoring, some of the immobilized MKs change shape, suggesting that they engage in the process of cytoskeletal reorganisation leading to platelet release. When anchored at a pillar and exposed to shear, an initially round MK can elongate and take the shape of beads on a thread, as shown in Fig. 2c, Supplementary Fig. S3a and Supplementary Video 2. When visible, the main cellular body can be either attached to the pillar or located further down the floating filament. MK morphology is deprived of the branched features observed in static cultures^12^. Although the shear rate is null at the back of an empty pillar, cells anchored at this location experience deformation. Supplementary Fig. S1 displays the wall shear rate on a 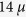
m diameter sphere placed behind a 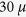
m diameter pillar. The shear rate measured on the sides of the sphere is of comparable magnitude with that on the sides of the pillars.

**Figure 3:**
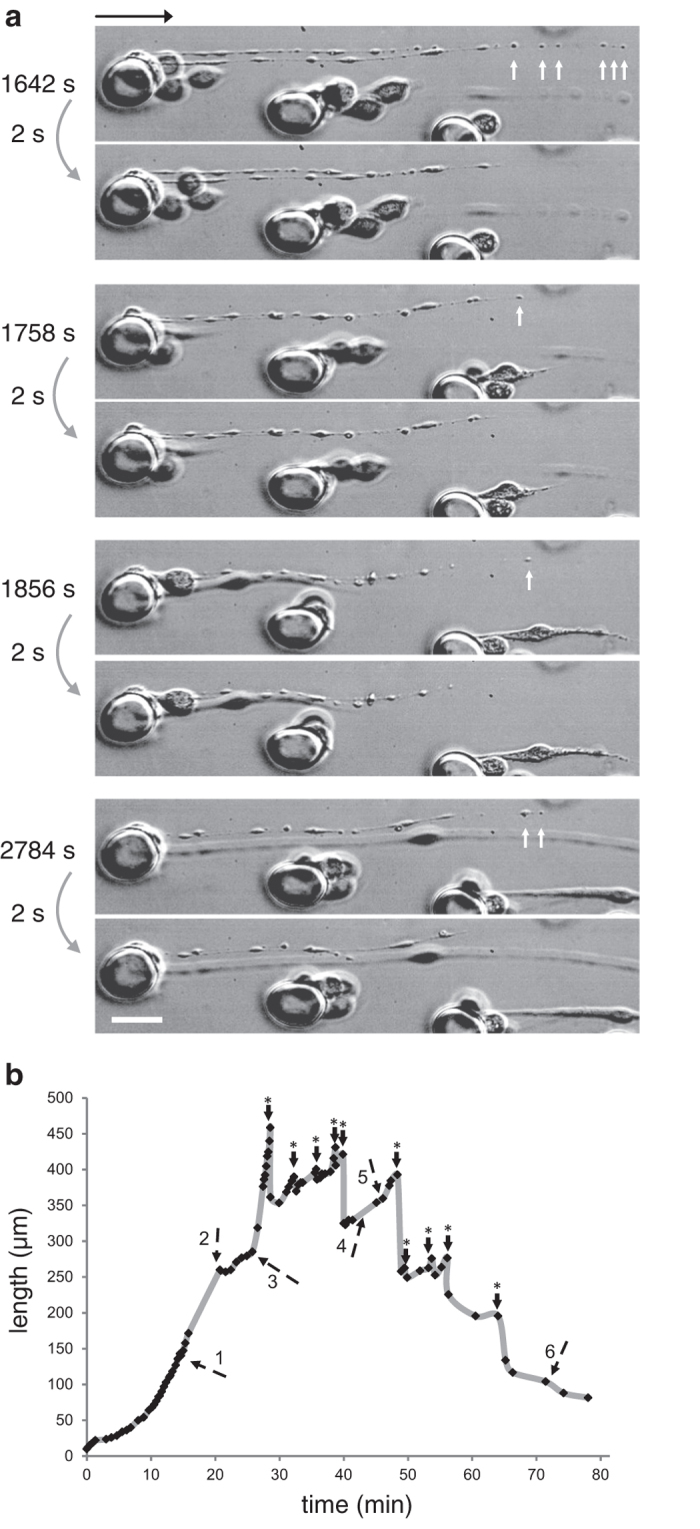
Elongation and rupture of MKs. (**a**) Time lapse observation of a single MK (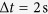). The origin of time is defined as the moment when the circulating MK is captured by a pillar. The first rupture occurs at *t* = 27 min, when a portion of string bearing 6 beads detaches from the rest of the cell. Other similar events occur at later times, involving one (*t* = 29 or 31 min) or several (*t* = 46 min) beads. Scale bar, 20 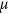
m. (**b**) Length of a megakaryocyte (n = 1). Stars (*) indicate fragmentation and numbers indicate different phases of the elongation process: (1) reorganization of proplatelets, (2) anchoring to a 2nd pillar, (3) dissociation from 1st pillar, (4) reorganization of proplatelets, (5) untangling of megakaryocyte segments (6) retraction of the residual chain. The time t = 0 min corresponds to the megakaryocyte anchorage to the first pillar. The sequence of pictures presented in panel (**a**) corresponds to snapshots taken between times depicted by arrows 3 and 5. The data are extracted from video 3.

We observed a plurality of MKs throughout their elongation process. Shortly after capture, the elongation velocity exhibits little variation (Supplementary Fig. S4). Even after a few platelets have been released, a stable elongation velocity may be measured between two ruptures (Fig. 3b). A mean speed of 25 ± 20 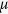
m/min (mean ± SD) was measured on 46 stable elongation phases from 25 elongating MKs obtained from 4 different umbilical cords.

In Fig. 2e, we show the time evolution of the density of elongated MKs, which starts increasing only after a delay of 5 to 10 min. Then, some of the captured MKs change their shape but the number of elongated MKs never reaches that of captured MKs. After 40 min perfusion, only 10 to 50% of trapped MKs exhibit elongations. At this time, close to the channel entrance, the density of elongated MKs decreases with time. We also notice that, at a given time, both densities decrease with the distance from channel entrance. This axial dependency has been observed for platelets during blood perfusions in VWF coated flow chambers^13^. Similar tendencies are observed with more concentrated perfusions, even in the case where clogs appear at the channel entrance (Supplementary Fig. S5).

### Cell fragmentation and platelet release

Figure 3a and Supplementary Videos 2, 3, and 4 illustrate the rupture process of an anchored MK, which starts approximately 20 minutes after capture. Its membrane repeatedly breaks between two beads, allowing the free extremity to be advected away by the flow, as shown in Supplementary Fig. S3b and Supplementary Video 4. The characteristic shape of beads on a string persists when the density of adherent cells increases. At high cell density, although the velocity field in the fluid around the obstacle is modified by the presence of adherent cells, captures, elongations and ruptures still occur as can be seen in Fig. 1c and Supplementary Videos 5 and 6.

The size of an elongated MK can reach several millimetres, which exceed by far the size of a microscope field when magnification is sufficient to observe platelets. However, in a few cases, we manage to keep track of the whole cell from capture to fragmentation and to visualise several successive rupture events (see Supplementary Videos 2 and 3). We can see in Fig. 3b that the elongation curve displays regions of constant slope separated by moments of pause or acceleration. The length of the MK sometimes suddenly decreases: this corresponds to the release of a proplatelet. Ruptures are usually preceded by a brief acceleration.

In Supplementary Video 3, we observed 11 ruptures per hour, and the volume of the released fragments corresponds to that of approximately 350 platelets. This is consistent with data from the literature: a human MK is expected to convert *in vivo* into 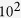 to 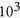 platelets^14^.

## Platelet characterisation

### Platelet yield

At the beginning and at the end of perfusion, cell suspensions are collected and examined with a hemocytometer. We define platelet-like particles (PLPs) as objects whose diameter is smaller than 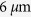. After 12 days of culture, MK suspensions contain many PLPs, usually a few times as many as MKs. To get rid of this background noise, we perform a centrifugation over a BSA gradient and collect a purified MK suspension. This allows a more faithful evaluation of platelet yield, and guarantees that platelets collected in the bioreactor effluent are indeed platelets produced on chip.

**Figure 4:**
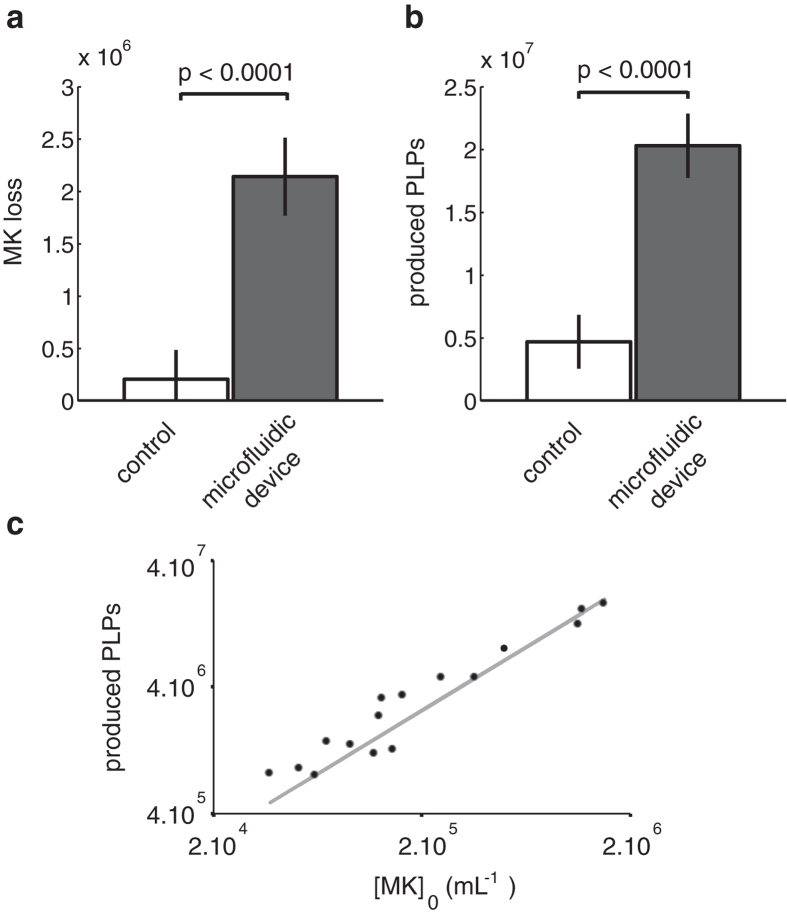
Counts of MK loss and produced platelets in the microfluidic device and in the control sample. (**a**) Variation of the number of MKs in the reservoir during a 2 h perfusion (20 mL, 5 chips). (**b**) Variation of the number of platelet-like particles 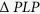 in the reservoir during a 2 h perfusion (20 mL, 5 chips). (**c**) Influence of the suspension concentration 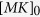 on the number 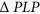 of released platelet-like-particles during a 2 h perfusion with a small volume of culture (6.7 mL). The curve can be fitted with a linear law (slope 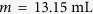, correlation coefficient 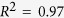).

As control, we use an extra reservoir filled with MK suspension, submitted to constant agitation and connected to the peristaltic pump, using the same tubing as the main sample, except that there is no microfluidic chamber in the circuit (control). In Fig. 4a,b we display the average MK loss and the produced platelets in the microfluidic device and the control sample (average on 8 experiments). The PLP amount increases in the control, presumably related to spontaneous fragmentation due to agitation and flow through the pump. However, the significant difference (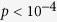) between control circulation and the one through the chips reveals that flowing through the microfluidic device enhances these spontaneous processes. We performed experiments using MK suspension increasing concentration (Fig. 4c). The number of produced PLPs increases linearly with MK concentration over a wide range, suggesting that the chip does not saturate.

We define platelet yield as the ratio between the number of PLPs created during the perfusion and the number of MKs injected in the chip. In this equation, 0 and f stand for initial and final conditions.


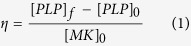


The supplementary Table 1 shows that the mean platelet yield measured at the end of a 2h perfusion is 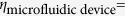 3.7 PLP/MK. In order to evaluate the role of the device in the fragmentation process, we also measure 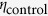 = 1.1 PLP/MK in the control. This indicates that although a fraction of platelet production can be accounted for by the mere circulation of the suspension through the tubes or agitation, the presence of pillars allows for a significant increase in platelet production. In order to increase the platelet number, washout experiments of the chamber with a buffer containing EDTA (Ethylenediaminetetraacetic acid) indicated an additional 10% increase of platelet recovery (data not shown).

One chip contains 170000 pillars. We have shown that the typical duration of the fragmentation process is around 20 minutes, so a perfusion lasting two hours should allow for the capture and fragmentation of over 1 million of MKs/chip. Considering the fact that several MKs are routinely seen attached to the same pillar, the potential of capture is even larger. The number of MKs injected in the microfluidic device in the experiments presenting in this manuscript is about 1 million/chip. The fact that not all of these MKs get captured can be explained by the heterogeneity of the cell suspension. In addition, separate experiments have shown that only a fraction of all MKs are CD41^+^CD42b^+^ and thus able to interact with VWF.

### Biological characterisation

**Figure 5:**
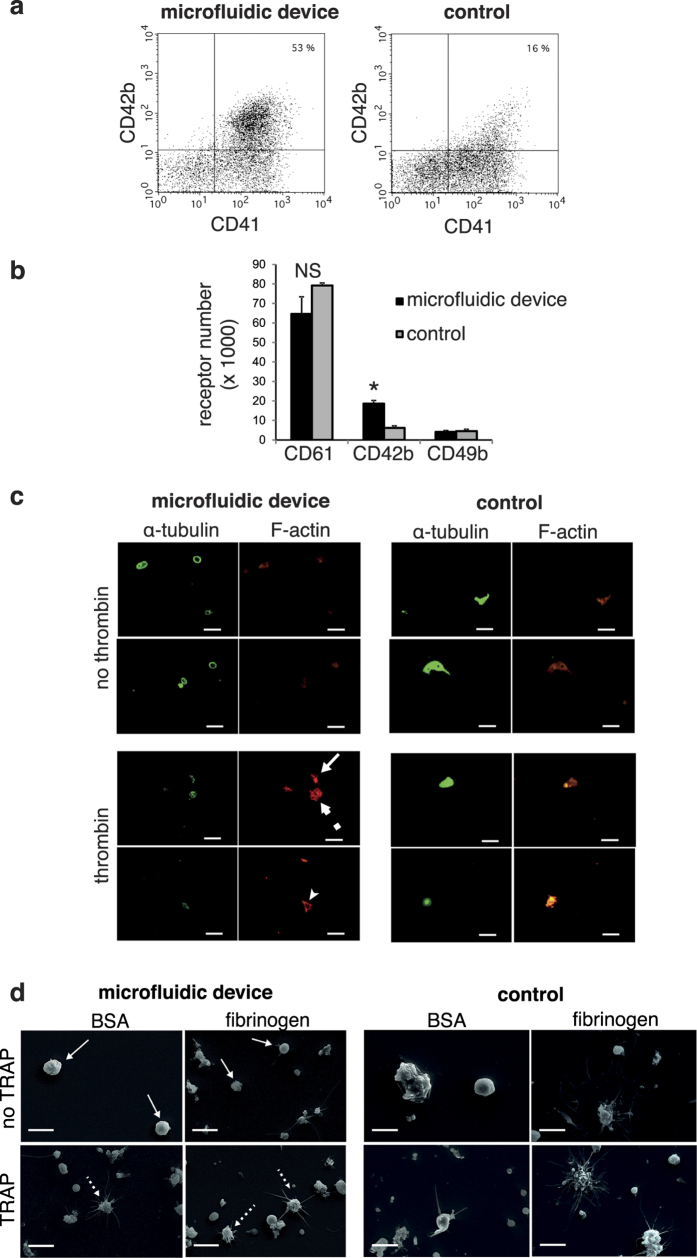
Characterisation of platelet morphology in samples collected at the exit of the microfluidic device and in the control sample. (**a**) Two-color flow cytometry analysis of platelets receptors, indicating the population of CD41^+^/CD42b^+^ platelets. (**b**) Single colour flow cytometry analysis of platelet receptor density, indicating the number of CD61, CD42b and CD49b receptors on the surface of platelets (n = 3). (**c**) Indirect immunofluorescence labelling with an anti-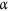-tubulin antibody, revealed by a secondary AlexaFluor488 goat anti-mouse antibody and AlexaFluor546 phalloidin for F-actin staining is performed in the absence (top panel) or presence (bottom panel) of thrombin. Circular tubulin staining, characteristic of unactivated platelets is seen in the samples collected at the exit of the fluidic device (top left), whereas larger fragments without circular tubulin staining are recovered in samples collected from the control (top right). Actin stress fibres (small arrow, full arrow indicate a filopod and dotted arrow a lamellipod) characteristic of activated platelets are seen in the samples collected at the exit of the fluidic device (bottom left), whereas larger elements without organised stress fibres staining are recovered in samples collected from the control (bottom right). Platelets are adherent to fibrinogen. Scale bar 5 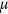
m. (**d**) Electron microscopy observations of platelets on BSA or fibrinogen coated surface, upon TRAP activation. Discoid round platelets are indicated with full white arrows and pseudopods with dotted arrows. Scale bar 5 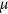
m.

**Figure 6:**
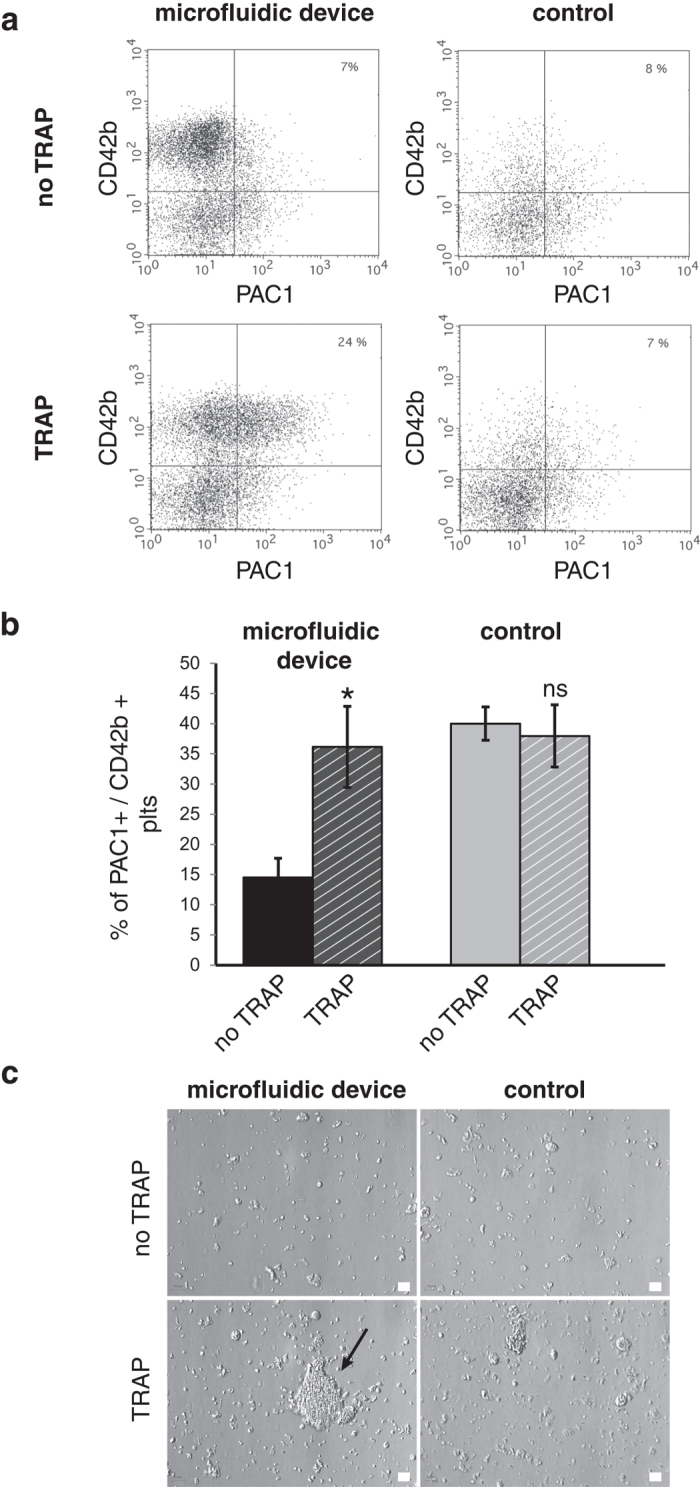
Characterisation of platelet activation. (**a**) Two-colour flow cytometry analysis of platelet receptors without or with activation with TRAP. Dot plots within the platelet gate indicate the population of PAC1 positive, CD42b^+^ platelets in the non stimulated (upper panels) and TRAP stimulated samples (lower panels) produced in the microfluidic device (left panels) vs control (right panels). (**b**) Quantification corresponding to the results presented in panel (**a**) (mean values for 4 independent experiments). (**c**) Aggregation in the presence of fibrinogen and CaCl_2_. Platelet aggregates are observed before (upper panels) or after (lower panels) activation with TRAP. The black arrow indicates a large aggregate visible in the sample collected at the exit of the fluidic device (lower left panel). Fragments recovered in the control samples do not aggregate in the presence of the agonist peptide (lower right panel). The scale bar represents 10 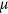
m.

Platelet morphology was obtained from flow cytometry analysis and gated according to FSC and SSC parameters of blood platelets (Fig. 5a,b): platelets produced in the device after 2h perfusion displayed features missing from the samples obtained from the control system. A well-defined population of CD41^+^ CD42b^+^ elements was identified in the microfluidic device sample, that was not found in the control (Fig. 5a). Moreover, as shown in Fig. 5b, the density of CD42b receptors was significantly higher in platelets produced by the microfluidic device than in control platelets (p < 0.05). Further characterisation of platelets produced in the chip involved tubulin/actin staining of the cytoskeleton. Non activated platelets from the microfluidic device display a tubulin ring characteristic of platelet discoid shape (Fig. 5c). Upon thrombin activation, actin organisation of lamellipods was visible only in platelets produced in the microfluidic device (Fig. 5c), indicating that actin filaments are reorganised as in thrombin-activated platelets isolated from blood. Both hallmarks of platelet functions were missing from the control samples. Scanning electron microscopy confirmed the presence of discoid round elements, which underwent pseudopodial and lamellipodial transition upon activation. Fragments without discoid shape were observed in samples from control (Fig. 5d). Interestingly, upon TRAP stimulation, platelets were able to undergo activation features similar to blood platelets. They were able to increase their levels of binding of the PAC1 monoclonal antibody, a finding that was absent in control platelets (Fig. 6a,b). Figure 6c shows a platelet aggregate in the sample recovered from passage through the microfluidic device. Again no such large aggregate was seen in platelets recovered in the control. Thus this report indicates for the first time that distinct features of platelets are observed in samples collected from exposure of megakaryocytes through a parallelised microfluidic device, that are missing from platelets recovered in control devoid of microfluidic chips. Scanning electron microscopy (Fig. 5d) shows the presence of filopodia and lamellipodia in TRAP activated platelets produced by the fluidic device, whereas these formations were not detected in the control samples. Finally, we checked that the produced platelets in the microfluidic device are able to bind to VWF coated on a surface (data not shown) and that their surface markers are comparable to the ones of platelets isolated from blood donors (Supplementary Fig. S7).

## Discussion

MKs cultured in static wells spread at the bottom surface in an non-isotropic way, exhibiting filaments with platelet-like buds, whose fragmentation takes place over about 12 h^12^. The resulting platelets are spread on the surface, indicating some level of activation. In the past decade, experiments showed that platelet production can be enhanced by exposing MKs to shear, paving the way for the development of fluidic devices dedicated to platelet production^6^,^7^. These systems attempt to model the physiological environment of the bone marrow, where MKs mature *in vivo*.

In some designs, MKs are pushed through narrow slits towards a model sinusoid where culture medium circulates with a high flow rate^3^,^8^. In an alternative bioreactor, MKs are seeded in an hydrogel containing a porous silk tube coated with growth factors^9^,^10^. MKs migrate towards the tube by chemoattraction. There, they produce elongations towards the lumen, where flowing medium collects fragments. Usually, the fragmentation process is slow, requiring 12 to 16 h to process a million cells. Thon *et al.* report a much shorter operation time (2 h, similar to that of our bioreactor), but the number of MKs processed in this time interval is limited to a few hundreds^8^.

In all these systems, an initial loading phase is followed by medium perfusion to achieve the desired shear rate. In ours on the contrary, like in the system described in^6^, the same flow is used to carry MKs into the chamber and to shear them. In such a single-flow chip, MKs arrive at capture sites in a progressive and more efficient manner. MKs continuously enter the production chamber even when the first pillars are already occupied by captured cells. It is therefore a major perspective for high cell throughput developments.

Platelets are here directly released in the bulk and continuously collected at the outlet without requiring washing steps. By avoiding contact between platelets and the VWF-coated surface, this bioreactor reduces the potential risk of rolling-induced platelet activation^15^. Proplatelets resulting from the rupture of an upstream MK can be captured downstream by another pillar. The whole fractal nature of platelet production process is thus recovered, in agreement with previous work showing that proplatelets are continuously fragmented as they circulate in blood^16^.

Our system also allowed to gain insight on microtubule dynamics during elongation. The elongation rate of mouse fetal liver MKs spread on a smooth surface has been measured to be 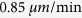
12. In the same study, the authors measure a wide variety of microtubules growth rates, with a maximal value of 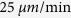. In our device, elongation rates can be as high as 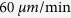, usually in the minute preceding a rupture. Such a velocity is probably the result of an interplay between microtubule-induced growth, presumably dynein-driven^17^, and flow-induced deformation. The largest velocities are observed for cells that already exhibit large deformations, whose filaments reach the regions of maximal shear rate. Since the region where elongation occurs colocalizes both with region of high surface shear rate and high extensional flow (Supplementary Fig. S1), we cannot conclude which of the mechanical forces is dominant in driving elongations.

Using video microscopy we can access information both at the scale of single cell and of the entire suspension. There, we observe that only a relatively small fraction of captured MKs deforms. This fraction depends on maturity of cells, including the proportion of CD41a^+^/CD42b^+^ cells (but possibly other markers), their cytoplasmic content, and hematopoietic stem cell origin.

Functional platelets need to be able to bind to endothelial VWF. Data presented in Fig. 5b demonstrate that platelets produced in our bioreactor express 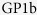 at a physiological level, contrary to platelets collected from some previous systems^3^. Also, because MK suspension was purified from most of its PLPs prior to perfusion, we can confirm without ambiguity that the functional platelets found in the effluent are the ones that were produced in the bioreactor. In most studies, the authors claim to reach a platelet yield of the order of a few platelets/MK. In our case, this was the proportion found in MK suspension before it was flown through the bioreactor. The largest value for platelet yield with human cells (20 to 30 platelets/MK) is found in reference^8^. This yield is estimated as the ratio of number of produced platelets by number of transformed MKs. Due to the limited number of narrow slits and the low flow rate, only a small number of MKs occupy the slits and release platelets. Moreover, platelets present in the initial suspension can flow through the slits much more easily than MKs, all the more so since about 1/3 of slits remain free of MK over the course of perfusion. Thus, it is likely that many platelets found in the effluent were actually preexisting in the suspension. Then, cytometry tests on the effluent cannot distinguish between platelets actually produced on chip and preexisting ones, which is a major limit of this approach.

We describe for the first time a device capable of simultaneously handling millions of MKs and leading to their fragmentation into platelets after a simple 2 hour perfusion of MK suspension. Bioreactor fabrication is fast and cheap since it involves only standard PDMS protocol and coating with one protein. In contrast to other platelet production methods in the literature, we evaluate platelet yield in a thorough manner, representing the actual platelet production during the flow experiment. We thus demonstrate that the bioreactor microstructure is accountable for a significant part of platelet production. Also, pillars foster elongations and fragmentation in the bulk, avoiding both platelet activation and platelet loss due to adhesion to the walls. Collected platelets are found to be functional, with a low level of initial activation, and sensitive to chemical activation. Platelet production is often regarded as the final stage of a maturation process that occurs in the bone marrow and the vascular niche. Our findings show that the last stages of fragmentation are best reproduced at shear rates much higher than the shear rate at the sinusoid wall in the bone marrow. This suggests that platelet generation should not be looked at as a local phenomenon confined to the vascular niche, but rather as a process involving several tissues, from the bone marrow to the capillary beds. In this extended platelet-generating organ, blood flow both serves as a transport mechanism and as a way to impose the shear forces required to elongate and fragment MKs.

## Methods

### Cell culture and differentiation


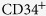
 cells were isolated from human umbilical cord blood (UCB) by an immunomagnetic technique (Miltenyi Biotec, Paris, France) as previously reported in^18^. This study was approved by the ethics committee/institutional review board “Assistance Publique-Hôpitaux de Paris, Cord Blood Bank, Saint-Louis Hospital, Paris, France” with women’s written informed consent, in accordance with the Declaration of Helsinki. 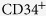 cells were cultured at 37 °C in 5% 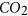 in complete medium consisting of Iscove Modified Dulbecco Medium (IMDM; GibcoInvitrogen, Saint-Aubin, France) supplemented with 15% BIT 9500 serum substitute (Stem Cells Technologies, Grenoble, France), 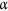-monothioglycerol (Sigma-Aldrich, Saint-Quentin Fallavier, France) and liposomes (phosphatidyl-choline, cholesterol and oleic acid; SigmaAldrich)^18^. Human recombinant stem cell factor (SCF, 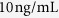; Miltenyi Biotec) and thrombopoietin peptide agonist AF13948 (TPO, 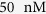) (see^6^) were added once at day 0 to the culture medium followed by addition of 20 nM TPO without SCF at day 7. Mature megakaryocytes obtained after 12–14 days of culture were diluted in complete medium to a concentration of  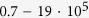 mL^−1^. Measured mean diameter 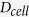 was found to be 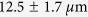. Removal of platelets formed during culture and immediately prior to shear exposure was performed by means of a Bovine Serum Albumin (BSA) gradient according to the methods reported in^19^. The concentration was then adjusted for perfusion. Unless otherwise specified, cells were perfused with a concentration between 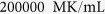 and 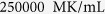.

### Microfluidic design

In order to define the optimal features for the production chamber, we use chips with two chambers used as channel inlet and outlet, connected with 8 parallel channels of equivalent hydraulic resistance and 4 different textures (each of them being replicated once). An example is shown in supplementary fig. S2A, where three channels with varying density of circular posts are compared with a smooth channel. The platelet producing chip comprises two large triangular inlet and outlet compartments, the height of each compartment being 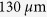. These dimensions limit sedimentation and avoid wall shear rate over 2000 s^−1^. These chambers are connected with 16 identical channels, whose S-shape allows for microscope observation of the whole system, while the total length of each channel is 17.3 cm. Channels are 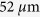 deep and textured on their straight parts, representing 77% of the total length. The radius of pillars is 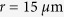. Unless otherwise specified, the distance between neighbouring pillars, from centre to centre, is 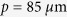. The angle between the main direction of the pillar array and that of the channel is chosen so that each MK encounter at least one pillar (10°). The height of the pillars is here equal to the depth of the channel. The shear rate in an empty channel and in the vicinity of a cell trapped behind a pillar are computed by numerical simulation (Comsol Multiphysics v3.9).

### Chip fabrication

Microfluidic chips are fabricated in polydimethylsiloxane (PDMS) using standard soft lithography techniques^20^. Briefly, a master is created by spin coating photoresist (SU8, Microchem, USA) on a on a silicon wafer and shining UV light through a mask. Wafers are developed after insolation. This process is repeated with a second layer of SU8 and a second mask, to obtain locally different heights at the inlet and outlet compartments. PDMS and curing agent (Sylgard) are mixed in a 10:1 ratio and cast on the master. After 2h at 70 °C the PDMS is cross linked and can be peeled off from the master. It is then activated with an oxygen plasma, together with a glass slide. Slide and chip are sealed and the chamber is immediately filled with a 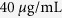 VWF (Wilfactin, LFB Biomedicaments, Les Ulis, France) solution before being placed at 4 °C for overnight incubation. To remove unbound VWF, channels are washed with Phosphate Buffer Saline (PBS) prior to experiments.

### Flow experiment

The reservoir containing the MK suspension is placed on an orbital shaker rotating at a moderate speed, to prevent cell sedimentation and ensure that the concentration in the suspension remains homogeneous during the experiment. This reservoir is connected to the perfusion chamber by two tubes plugged in the chamber inlet and outlet. A continuous circulation is achieved by the means of a peristaltic pump (Watson-Marlow). Several chips may be used in parallel for large volume experiments (typically 1 chip/5 mL suspension). The chip is placed on the stage of an inverted microscope (DMI6000 B, Leica Microsystems GmbH, Germany). A high-speed camera (Fastcam SA3, Photron, USA) allows to evaluate cell density and shape at different locations on the chip. At the end of the experiment, the suspension in the reservoir is collected. The concentration of MKs and PLPs is assayed by manual counts on a Malassez slide. For each set of parameters (MK concentration, number of chips in parallel, suspension volume), at least 5 independent experiments are performed.

### Characterization of produced platelets

Expression of CD41 and CD42b antigens was characterized using a flow cytometer BD Fluorescence Activated Cell Sorter (FACS) Calibur (BD Biosciences, Le Pont de Claix, France). Platelets were incubated with fluorescein isothiocyanate (FITC)-conjugated anti-human CD41 (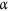
IIb) and R-phycoerythrin (PE)-conjugated anti-human CD42b (GPIb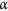) (both from Beckman Coulter, Villepinte, France) during 20 minutes at room temperature. Controls were performed using FITC mouse IgG1 (Beckman Coulter), PE mouse IgG1 (Beckman Coulter). Single color flow cytometric analysis of platelet receptors was performed using the GP screen assay (Biocytex, Marseille, France). The number of antigenic sites is determined by converting the fluorescence intensity into corresponding numbers of monoclonal antibodies bound per platelet based on a calibrated bead standard curve. The activated form of 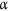
IIb
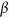
3 (FITC anti-PAC1) was analysed by flow cytometry with PE anti human CD42b. The activation of the platelet suspension, not submitted to BSA gradient, was obtained in the presence of an agonist peptide of the PAR-1 thrombin receptor TRAP (20 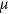
M) diluted in a tyrode buffer without CaCl_2_.

Fibrinogen adhesion assay and epifluorescence characterisation were performed redundancy in as previously reported in^6^, except that activation was obtained in the presence of thrombin (1 U/mL).

Tubulin/actin staining of the cytoskeleton and PAC-1 immunofluorescence were studied on adhered platelets. Ibidi chambers (Biovalley, Marne-la-vallée, France) were first coated with fibrinogen (20 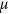
g/mL during 2h) and rinsed with tyrode buffer. For PAC-1 immunofluorescence, platelets (with and without TRAP activation) are deposited on the coated chambers for adhesion 20 min at 37 °C. The supernatant is removed and replaced by PAC-1 solution (10 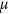
g/mL in tyrode buffer without CaCl_2_) at room temperature for 10 min. After tyrode rinsing, cells were fixed with 4% paraformaldehyde (PFA) during 15 min, then permeabilised for 5 min with 0.1% Triton X-100 in PBS and non-specific binding blocked in 2% PBS-BSA during 30 min. After that, fixed cells were incubated with AlexaFluor-488 goat anti-mouse IgM (20 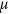
g/mL) during 45 min at 37 °C, rinsed then incubated with AlexaFluor-546 Phalloidin (5U/mL). The coverslips were then washed and incubated with Hoechst before mounting in DABCO.

Epifluorescence was analysed at 494 nm and 522 nm (absorption and emission, respectively), using the Axio Observer D1 fluorescence optical microscope (Zeiss, Le Pecq, France) with a high-resolution bioimaging platform (EMCCD MGi Plus Qimaging Rolera camera, Roper Scientific, Evry, France) for clog analysis and QIclick F-CLR-12 colour 12 BIT, Qimaging (Roper Scientific, Evry, France) for other immunofluorescence imaging.

Aggregation was performed using a dual-channel Whole Blood/Optical Lumi-Aggregometer (Model 700 Chronolog Corporation). Collected platelets were isolated using a BSA gradient and suspended in a tyrode buffer before adding Fibrinogen (200 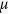
g/mL). After 20 min incubation at room temperature, CaCl_2_ was added (2 mM, 10 min at 37 °C) before TRAP activation of platelets (10 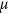
M) and agitation during 5 minutes. Platelets were then fixed using 2% PFA and deposited on LabTek chambers at 4 °C before observation.

For electron microscopy observations, platelets were isolated using a BSA gradient (described above) and stimulated or not with TRAP 10 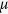
M (Bachem, Bubendorf, Switzeland). Cells were firstly fixed on Fibrinogen or BSA-coated coverslips with 2.5% glutaraldehyde and 2% paraformaldehyde for 45 minutes at room temperature. Samples were then washed thrice with 0.1M sodium cacodylate buffer and kept overnight at 4 °C, followed by a second fixation in 1% Osmium tetroxide for 30 minutes. After washing, cells were finally dehydrated with an Ethanol gradient and hexamethyldisilazane (all reagents for sample preparation were purchased from Sigma Aldrich (Saint-Quentin Fallavier, France)). Following dehydration, cells were sputter-coated with gold-palladium using JEOL JFC-1300 auto fine coater (Jeol, Tokyo, Japan). Electron microscopy analysis was performed on a JSM-6510 LV scanning electron microscope, (Jeol, Peabody, USA) at an accelerated voltage of 10 kV and working distance of 11 mm.

## Additional Information

**How to cite this article**: Blin, A. *et al.* Microfluidic model of the platelet-generating organ: beyond bone marrow biomimetics. *Sci. Rep.* **6**, 21700; doi: 10.1038/srep21700 (2016).

## Supplementary Material

Supplementary Video 1

Supplementary Video 2

Supplementary Video 3

Supplementary Video 4

Supplementary Video 5

Supplementary Video 6

Supplementary Information

## References

[b1] Doshi N. *et al.* Platelet mimetic particles for targeting thrombi in flowing blood. Adv. Mater. 24, 3864–3869 10.1002/adma.201200607 (2012). 22641451 PMC3483800

[b2] Takayama N. *et al.* Generation of functional platelets from human embryonic stem cells *in vitro* via ES-sacs, VEGF-promoted structures that concentrate hematopoietic progenitors. Blood 111, 5298–5306 10.1182/blood-2007-10-117622 (2008). 18388179

[b3] Nakagawa Y. *et al.* Two differential flows in a bioreactor promoted platelet generation from human pluripotent stem cell-derived megakaryocytes. Exp. Hematol. 41, 742–748 10.1016/j.exphem.2013.04.007 (2013). 23618622

[b4] Feng Q. *et al.* Scalable generation of universal platelets from human induced pluripotent stem cells. Stem Cell Reports 3, 817–831 10.1016/j.stemcr.2014.09.010 (2014). 25418726 PMC4235139

[b5] Cid J. & Lozano M. Platelet dose for prophylactic platelet transfusions. Expert Rev. Hematol. 3, 397–400 10.1586/ehm.10.36 (2010). 21083030

[b6] Dunois-Lardé C. *et al.* Exposure of human megakaryocytes to high shear rates accelerates platelet production. Blood 114, 1875–1883 10.1182/blood-2009-03-209205 (2009). 19525480

[b7] Junt T. *et al.* Dynamic visualization of thrombopoiesis within bone marrow. Science 317, 1767–1770 10.1126/science.1146304 (2007). 17885137

[b8] Thon J. N. *et al.* Platelet bioreactor-on-a-chip. Blood 124, 1857–1867 10.1182/blood-2014-05-574913 (2014). 25606631 PMC4168343

[b9] Pallotta I., Lovett M., Kaplan D. L. & Balduini A. Three-dimensional system for the *in vitro* study of megakaryocytes and functional platelet production using silk-based vascular tubes. Tissue Eng. Part C Methods 17, 1223–1232 10.1089/ten.tec.2011.0134 (2011). 21895494 PMC3226422

[b10] Di Buduo C. A. *et al.* Programmable 3D silk bone marrow niche for platelet generation *ex vivo* and modeling of megakaryopoiesis pathologies. Blood 125, 2254–2264 10.1182/blood-2014-08-595561 (2015). 25575540 PMC4383799

[b11] Chang W. C., Lee L. P. & Liepmann D. Biomimetic technique for adhesion-based collection and separation of cells in a microfluidic channel. Lab Chip 5, 64–73 10.1039/B400455H (2005). 15616742

[b12] Patel S. R. *et al.* Differential roles of microtubule assembly and sliding in proplatelet formation by megakaryocytes. Blood 106, 4076–4085 10.1182/blood-2005-06-2204 (2005). 16118321 PMC1895246

[b13] Sakariassen K. S. & Baumgartner H. R. Axial dependence of platelet-collagen interactions in flowing blood. upstream trombus growth impairs downstream platelet adhesion. Arterioscler. Thromb. Vasc. Biol. 9, 33–42 10.1161/01.ATV.9.1.33 (1989). 2783548

[b14] Thon J. N. *et al.* Cytoskeletal mechanics of proplatelet maturation and platelet release. J. Cell Biol. 191, 861–874 10.1083/jcb.201006102 (2010). 21079248 PMC2983072

[b15] Ruggeri Z. M. Von Willebrand factor, platelets and endothelial cell interactions. J. Thromb. Haemost. 1, 1335–1342 10.1046/j.1538-7836.2003.00260.x (2003). 12871266

[b16] Poirault-Chassac S. *et al.* Terminal platelet production is regulated by von Willebrand factor. PLoS ONE 8, e63810 10.1371/journal.pone.0063810 (2013). 23737952 PMC3667798

[b17] Bender M. *et al.* Microtubule sliding drives proplatelet elongation and is dependent on cytoplasmic dynein. Blood 125, 860–868 10.1182/blood-2014-09-600858 (2015). 25411426 PMC4311231

[b18] Poirault-Chassac S. *et al.* Notch/Delta4 signaling inhibits human megakaryocytic terminal differentiation. Blood 116, 5670–5678 10.1182/blood-2010-05-285957 (2010). 20829371

[b19] Robert A., Cortin V., Garnier A. & Pineault N. Megakaryocyte and platelet production from human cord blood stem cells. In Gibbins J. M. & Mahaut-Smith M. P. (eds) Platelets and Megakaryocytes, vol. 788 of *Methods in Molecular Biology*, 219–247, Springer: New York, 10.1007/978-1-61779-307-3_16 (2012). 22130711

[b20] Whitesides G. M., Ostuni E., Takayama S., Jiang X. & Ingber D. E. Soft lithography in biology and biochemistry. Annu. Rev. Biomed. Eng. 3, 335–373 10.1146/annurev.bioeng.3.1.335 (2001). 11447067

