# Supplementary material for: Microfluidic model of the platelet-generating organ: beyond bone marrow biomimetics
Source: Sci Rep. 2016 Feb 22;6:21700. doi: 10.1038/srep21700 (PMC4761988; doi:10.1038/srep21700)
Supplement: Supplementary Information [file srep21700-s7.pdf]

Microfluidic model of the platelet-generating organ: beyond bone marrow biomimetics

Antoine Blin[+], Anne Le Goff[+], Aurélie Magniez, Sonia Poirault-Chassac, Bruno Teste, Géraldine Sicot, Kim Anh Nguyen, Feriel S. Hamdi Mathilde Reyssat[*], Dominique Baruch

+ : these authors contributed equally to this work
* Corresponding author (mathilde.reyssat@espci.fr)

Supplementary information

1 Supplementary table

	chips	volume (ml)	$[\text{MK}]_0$ (10^5/ml)	$[\text{MK}]_f$ (10^5/ml)	$[\text{PLP}]_0$ (10^5/ml)	$[\text{PLP}]_f$ (10^5/ml)	η
microfluidic device	5	20	2.58 ± 0.12	1.59± 0.09	2.78±0.45	12.30±1.16	3.7
control	0	20	2.41 ± 0.11	2.29±0.12	2.81±0.41	5.35±0.69	1.1

Table 1: MK and PLP counts before and after a 2-hour perfusion. Data are presented as mean ± SEM, with $n = 8$ for experiments with 5 chips, and $n = 12$ for experiments without chip. Data in the figure 4a and 4b panels correspond to experiments presented in this table with 5 chips (microfluidic device) or without chip (control). η is the platelet yield defined by equation (1). 0 and f stand for initial and final conditions.

2 Supplementary videos

Video 1. This video illustrates the capture and the elongation of a MK in a bioreactor. The first part of the video is a movie recorded with a high-speed camera. It illustrates the capture of a MK onto a pillar. The MK is advected by the flow before encountering a VWF coated pillar. Once captured by the pillar, the MK rolls around the pillar by translocation. The second part of the video is a time-lapse recording of a MK elongation. The second pillar starting from the left of the frame captures a MK. The MK is

attached downstream of the pillar after translocating around it as explained in the first part of the video. The MK undergoes fluid shear and starts elongating. After 15 min, the elongation starts modifying its shape to form beads on a string (right side of the frame). In both parts of the video, the white arrow represents the mean flow direction. The scale bars represent 20 μm.

Video 2. This video is a time-lapse recording, illustrating platelet and proplatelet releases from an elongated MK. The MK is attached downstream of the left pillar by the mechanism explained in Video 1, and has already started elongating at the beginning of the video. Five ruptures of the beads-on-a-string shape elongation occur and are shown with the triangular arrows. Platelets and proplatelets are released in the flow at high speed explaining their disappearance when a rupture occurs. The white arrow represents the mean flow direction. The scale bar represents 20 μm.

Video 3. This video is a time-lapse recording illustrating platelet and proplatelet releases from a single MK corresponding to the figure 3. The capture of the MK and the subsequent ruptures of the elongated MK are shown by triangular white arrows. The top-left white arrow represents the mean flow direction. The scale bar represents 20 μm.

Video 4. This video is a high-speed recording a platelet release in the flow. After the rupture, the left part of the elongation stays attached to a captured MK (not seen in the frame) whereas the right part of the elongation is dragged by the flow, following the streamlines. The white arrow represents the mean flow direction. The scale bar represents 20 μm.

Video 5. This video is a time-lapse recording of a parallelized platelet and proplatelet production in the bioreactor. It illustrates a part of a single channel of the chip where MKs are simultaneously undergoing elongation and rupture. Different ruptures are indicated by red transparent ellipses. The black arrow represents the mean flow direction. The scale bar represents 100 μm.

Video 6. This video is a time-lapse recording of a parallelized platelets and proplatelets production in the bioreactor. It illustrates the capture of MKs from the beginning of the experiment (no MK) and shows captures, elongations and ruptures of MKs in three parallel channels of the bioreactor. The scale bar represents 200 μm.

3 Supplementary figures

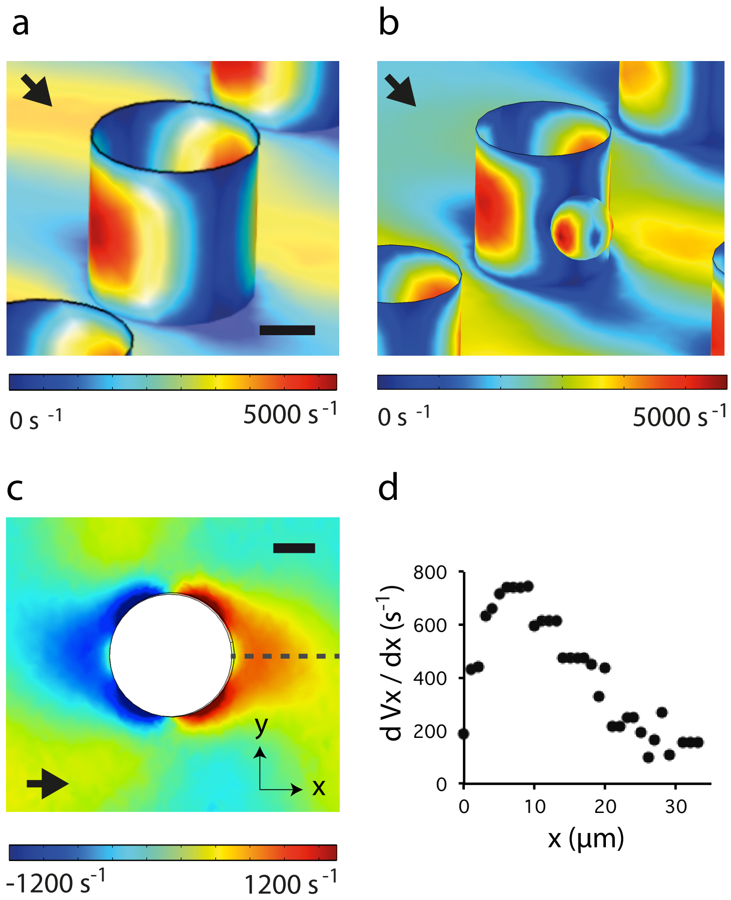

Figure S1: Numerical simulations. (a) 3D numerical simulation of the surface shear rate around an empty pillar. (b) 3D numerical simulation of the surface shear rate around a pillar with a 14 μm diameter sphere anchored downstream of the obstacle. The black arrow indicates the mean flow direction. The scale bar represents 10 μm. (c) 3D numerical simulation of the quantity dVx/dx in the fluid around a pillar, top view of a cutting plane at the mid-height of the channel. The black arrow indicates the mean flow direction. The dash line represents the direction of the cut plotted on (d). The scale bar represents 10 μm. (d) Evolution of dVx/dx as a function of x on the dash line on (c).

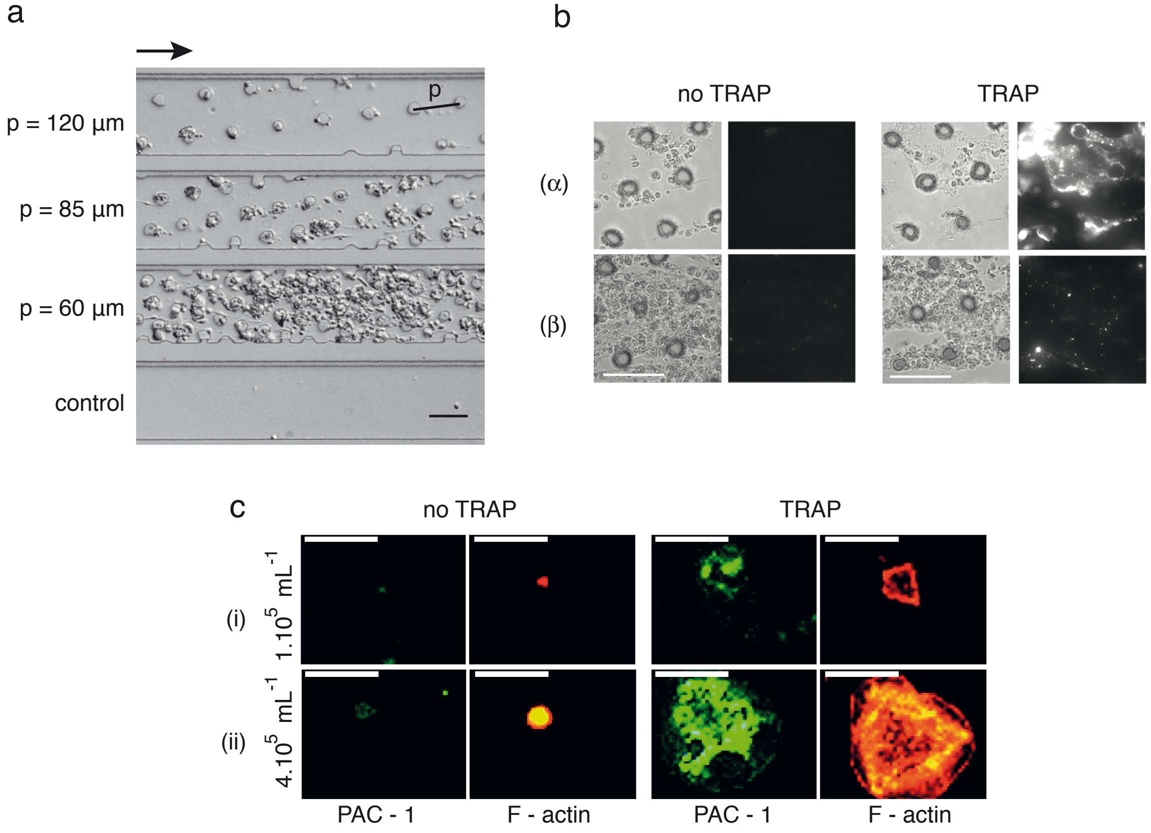

Figure S2: Characterization of cell clogging. (a) Photography of four channels textured with varying pillar density. The different channels are respectively patterned with interpillar distance p = 120 μm, 85 μm and 60 μm. The last channel is a negative test. Perfusion of 100 000 MK/ml is made in each channel. The image is taken after 50 min of experiment. Clogging occurs in the third channel, with the lowest value of $p = 60$ μm. The scale bar represents 100 μm. Similar clogs can be observed when the concentration of MK suspension increases ([MK] $\geq$ 200 000/ml for $p = 85$ μm). (b) To determine whether cells entangled in clogs are activated, we examined the activation state of αIIbβ3 receptor using fibrinogen AlexaFluor-488 in the microfluidic device in the presence of clogs (400 000 MK/ml) after a perfusion of 2 hours. Cells were stimulated using 50 μM TRAP or not stimulated (no TRAP) and stained with Alexa Fluor-488 fibrinogen. Fluorescence indicates the activated state of cells. (α) and (β) represent two distinct configurations: absence or presence of clogs. Cells entangled in the formed clogs are not fluorescent if not stimulated with TRAP indicating that the cells forming a clog are not activated. Scale bar: 100 μm. (c) On the other hand, produced platelets were collected at the outlet of the microfluidic device after a perfusion of 2 hours with 100 000 MK/ml(i) and 400 000 MK/ml (ii) and their function was controlled by indirect immunofluorescence labeling with a PAC-1 antibody, revealed by a secondary AlexaFluor488 anti-mouse antibody and AlexaFluor546 phalloidin for F-actin staining, and performed in the absence (left panels) or presence (right panels) of TRAP. Scale bar: 5 μm. This results show that the platelets produced from the diluted and concentrated conditions present the same profile; they are not activated but stay functional. In conclusion, even if MKs cluster into clogs, they are not activated in them and produce platelets that are not activated and stay functional.

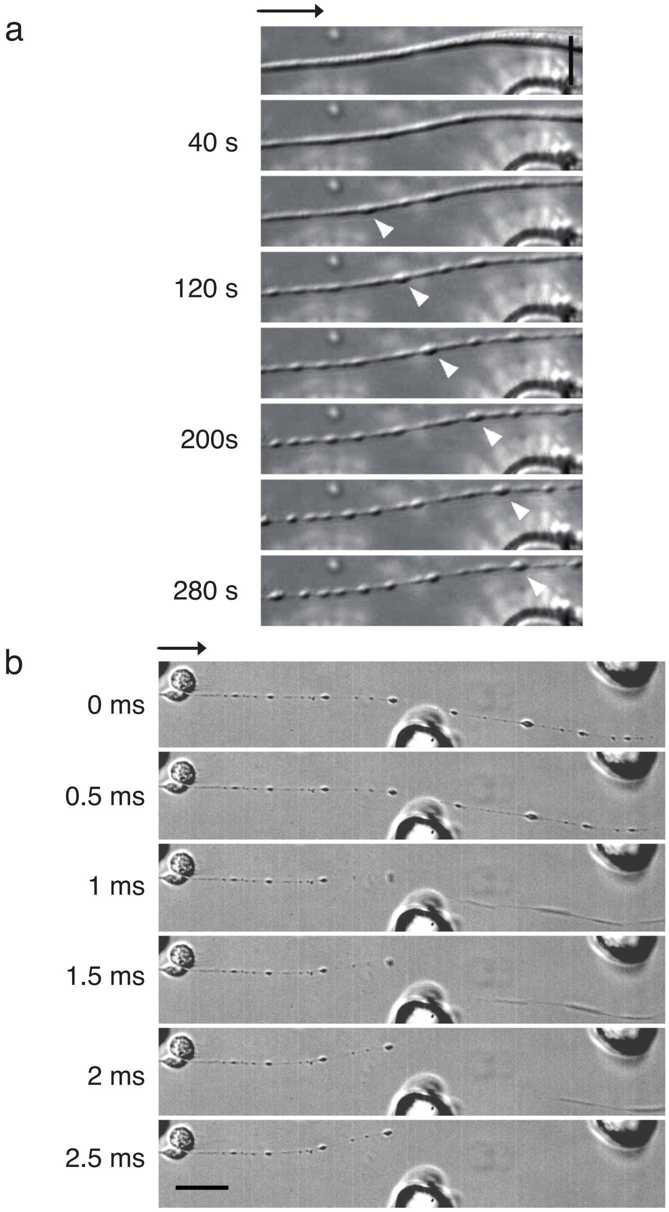

Figure S3: Optical observation of platelet formation and release. (a) Time lapse observation ($\Delta t = 40$ s) of the surface of an elongating MK. The initially smooth cylinder takes the shape of a necklace with round beads separated by thin filaments. Scale bar: 30 μm. (b) Time lapse observation ($\Delta t = 0.5$ ms) of a fragment released in the bulk by an elongated MK. Scale bar: 30 μm.

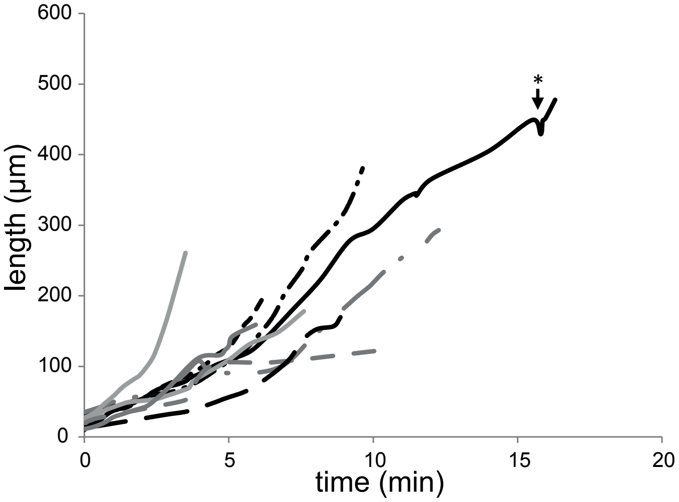

Figure S4: Length of elongating megakaryocytes as a function of time elapsed after capture. The star indicates a fragmentation.

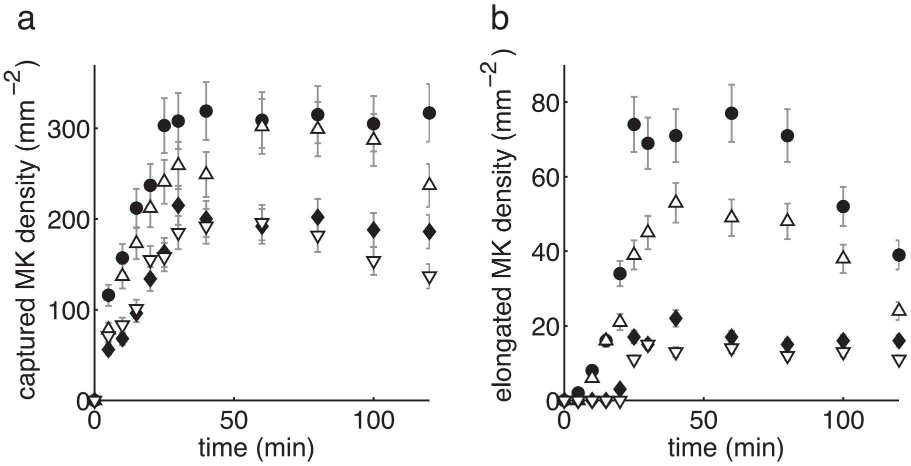

Figure S5: (a) Surface density of captured MKs as a function of time during a perfusion of a 400 000 MK/ml suspension through 5 chips, measured at different positions: $x = 10$ mm (•), $x = 20$ mm (△), $x = 60$ mm (⧫), $x = 120$ mm(▽). At $x = 10$ mm, due to apparition of clogs, no density measurement could be performed. Counts were performed by two experimentalists and represented as (mean ± SD). (b) Surface density of elongated MKs as a function of time during a perfusion of a 400 000 MK/ml suspension through 5 chips, for the same positions.

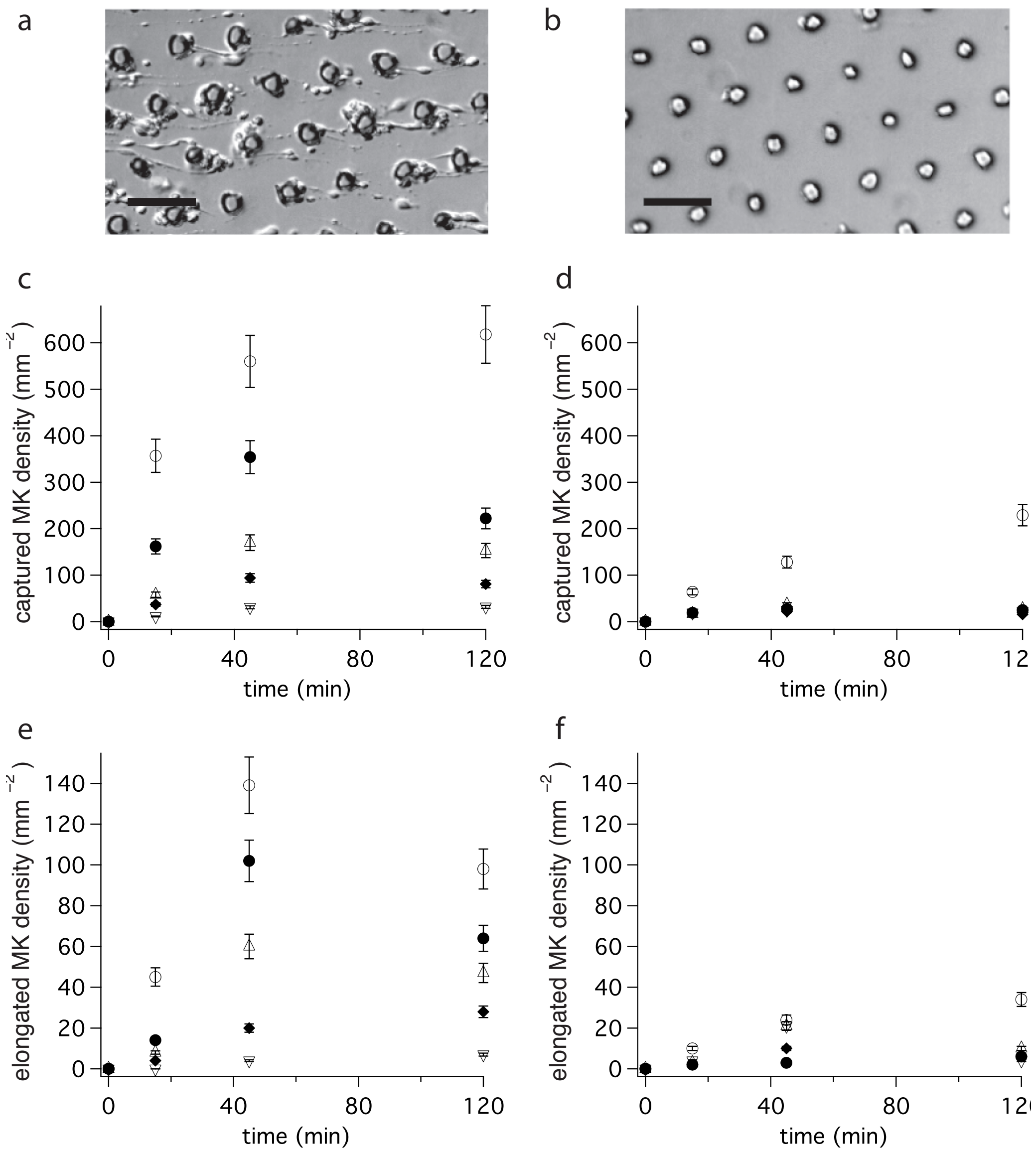

Figure S6: Comparaison of the capture and elongation of MKs on pillars coated by VWF (von Willebrand Factor) or BSA (Bovin Serum Albumin). (a, b) Images of the chips taken at x= 1mm after 30 minutes of perfusion in the VWF (a) and the BSA (b) coated devices. (c, d) Surface density of captured MKs as a function of time in the VWF (c) and the BSA (d) coated devices during a perfusion of a 200 000 MK/ml suspension through 1 chip (volume of perfusion = 6.7ml) measured at different positions: $x = 0$ mm (○), $x = 10$ mm (●), $x = 20$ mm (△), $x = 40$ mm (♦), $x = 120$ mm(▽). (e, f) Surface density of elongated MKs as a function of time for the same conditions on the VWF (e) and the BSA (f) coated devices.

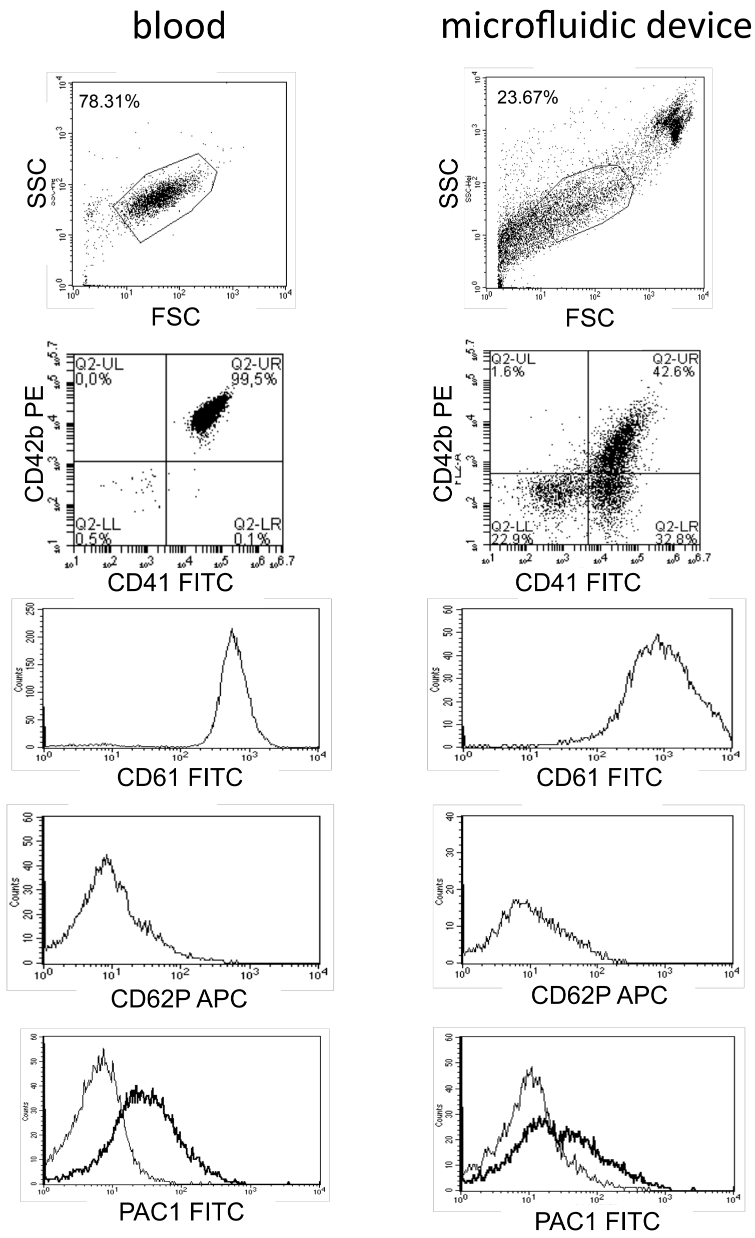

Figure S7: Comparison of fresh washed platelets isolated from blood with cells collected at the exit of the microfluidic device. The FSC/SSC dot plot of the isolated platelets from blood allows to draw a platelet gate, which is applied to calculate the % of PLP in the cells collected at the exit of the microfluidic device. Fluorescence intensity of CD61, the $\beta 3$ subunit of the αIIb$\beta 3$ receptor, is shown and was obtained using the GPscreen receptor density assay (Biocytex) depicted in Figure 5b. This quantitative assay allows to determine the expression of CD61 at the surface of platelets produced in the microfluidic device (64 633 $\pm$ 8 789 receptor/platelet; 3 experiments). This value compares with the density provided by the manufacturer for a large panel of normal blood platelets (53 000 $\pm$ 12 000). Expression of CD62P at the surface of platelets produced in the microfluidic device is 35.7% vs 17.8% for blood platelets. PAC1 antigen binding depicts the conformational change of αIIb$\beta 3$ receptor following platelet activation with 10 μM TRAP. Bold line depicts the platelet response to activation, whereas the thin line shows the amount of PAC1-FITC fluorescence in the absence of activation. Values are 58.7% and 37.6% positive platelets respectively for blood platelets and for platelets produced in the microfluidic device.
